# Estimating dengue under-reporting in Puerto Rico using a multiplier model

**DOI:** 10.1371/journal.pntd.0006650

**Published:** 2018-08-06

**Authors:** Manjunath B. Shankar, Rosa L. Rodríguez-Acosta, Tyler M. Sharp, Kay M. Tomashek, Harold S. Margolis, Martin I. Meltzer

**Affiliations:** 1 Division of Preparedness and Emerging Infections, National Center for Emerging and Zoonotic Infectious Diseases, Centers for Disease Control and Prevention, Atlanta, GA, United States of America; 2 Dengue Branch, Division of Vector-Borne Diseases, National Center for Emerging and Zoonotic Infectious Diseases, Centers for Disease Control and Prevention, San Juan, Puerto Rico; Fundacao Oswaldo Cruz, BRAZIL

## Abstract

Dengue is a mosquito-borne viral illness that causes a variety of health outcomes, from a mild acute febrile illness to potentially fatal severe dengue. Between 2005 and 2010, the annual number of suspected dengue cases reported to the Passive Dengue Surveillance System (PDSS) in Puerto Rico ranged from 2,346 in 2006 to 22,496 in 2010. Like other passive surveillance systems, PDSS is subject to under-reporting. To estimate the degree of under-reporting in Puerto Rico, we built separate inpatient and outpatient probability-based multiplier models, using data from two different surveillance systems—PDSS and the enhanced dengue surveillance system (EDSS). We adjusted reported cases to account for sensitivity of diagnostic tests, specimens with indeterminate results, and differences between PDSS and EDSS in numbers of reported dengue cases. In addition, for outpatients, we adjusted for the fact that less than 100% of medical providers submit diagnostic specimens from suspected cases. We estimated that a multiplication factor of between 5 (for 2010 data) to 9 (for 2006 data) must be used to correct for the under-reporting of the number of laboratory-positive dengue inpatients. Multiplication factors of between 21 (for 2010 data) to 115 (for 2008 data) must be used to correct for the under-reporting of laboratory-positive dengue outpatients. We also estimated that, after correcting for underreporting, the mean annual rate, for 2005–2010, of medically attended dengue in Puerto Rico to be between 2.1 (for dengue inpatients) to 7.8 (for dengue outpatients) per 1,000 population. These estimated rates compare to the reported rates of 0.4 (dengue outpatients) to 0.1 (dengue inpatients) per 1,000 population. The multipliers, while subject to limitations, will help public health officials correct for underreporting of dengue cases, and thus better evaluate the cost-and-benefits of possible interventions.

## Author summary

The number of global cases of dengue has increased an estimated 30-fold from 1962 to 2012, and two-fifths of the world’s population are thought to be at risk for dengue. It has been recently estimated that the global incidence of dengue is between 50 and 100 million cases per year. These estimates of burden and impact are, however, are not considered very reliable. It has been previously established and reported that there is notable under-reporting of clinical cases of dengue, even those who sought medical treatment. This includes under-reporting of those hospitalized with laboratory-confirmed dengue. This lack of reliable estimates hampers efforts of public health officials in determining the of burden of disease and the costs-and-benefits of potential interventions. We estimated that multiplication factors ranging from 5 to 9 must be used to correct for under-reporting of laboratory-positive dengue inpatient cases reported to public health officials in Puerto Rico. Multiplication factors ranging from 21 to 115 must be used to correct for the underreporting of laboratory-positive dengue outpatients. Our results illustrate the need for, and thus potential benefits of, using our methodology to estimate the degree of under-reporting in passive dengue systems during epidemic and non-epidemic years.

## Introduction

Dengue is a mosquito-borne viral illness that represents a major public health problem in all tropical and subtropical countries. Dengue incidence has increased an estimated 30-fold from 1962 to 2012, and two-fifths of the world’s population is thought to be at risk for dengue [1, 2]. It has been recently estimated that the global incidence of dengue is between 50 and 100 million cases per year [3]. Approximately 15% of the worldwide burden of dengue occurs in the Americas [4].

Dengue is endemic in Puerto Rico and has been a reportable condition for several decades [5]. Incidence of medically attended, clinically suspected cases of dengue has been monitored in Puerto Rico, since the late 1960s, by the Passive dengue surveillance system (PDSS). This system is operated by the Puerto Rico Department of Health (PRDH) and Centers for Disease Control and Prevention Dengue Branch (CDC-DB) [5–7]. In recent years, PDSS recorded dengue epidemics in 1998, 2007, 2010 and 2012–2013; the latter with 29,386 suspected dengue cases, of which approximately 54% were laboratory confirmed as dengue [8, 9]. However, like all passive surveillance systems, PDSS is subject to under-reporting of medically attended cases. Previous estimates indicated that for every suspected medically attended dengue case reported to PDSS, 10–27 additional cases occurred but were not reported [10–13]. Reasons for under-reporting included the need to obtain an acute and convalescent serum specimen to make an accurate laboratory diagnosis. This was difficult to implement and resulted in a substantial portion of suspected dengue cases being classified as indeterminate. In addition, non-hospitalized medically attended (i.e., outpatient) dengue cases were reported less frequently than hospitalized cases, and the frequency of reporting varied by region, type of healthcare facility and healthcare provider.

Estimating the degree of dengue under-reporting from standard passive surveillance systems is critical for making robust estimates of the true disease burden. Several approaches have been used to estimate the degree of dengue under-recognition and/or under-reporting. Wichmann et al. used childhood cohort studies to estimate the degree of under-reporting for national surveillance systems in Thailand and Cambodia [14]. A capture-recapture approach was used by Dechant et al to estimate the degree of under-reporting for inpatients by comparing passive surveillance data to data submitted by infection control nurses in Puerto Rico during 1991–1995[10]. Similarly, others have compared inpatient and emergency room hospital administrative data to surveillance data [15, 16] or used expert opinion to estimate under-reporting of hospitalized dengue cases [17, 18]. Since 2007, reporting by infection control nurses in Puerto Rico has ceased, and there is a need to update the multiplier estimates. In this analysis, we estimate the degree of under-estimation of medically attended dengue cases in Puerto Rico. Our model gives both point estimates of the multiplier, the uncertainty around them, and includes the effect on underreporting due to test sensitivity and number of indeterminate cases. We also provide separate estimates for inpatients and outpatients. Our estimates of multipliers will help public health officials correct for underreporting of dengue cases and thus enable them to evaluate better the cost-and-benefits of possible interventions.

## Methods and materials

### Ethics statement

This study underwent institutional review at the CDC and was determined to be public health practice (evaluation of an ongoing surveillance system) and not research; as such, we obtained a waiver from CDC’s Institutional Review Board approval.

### Model description

**Fig 1:**
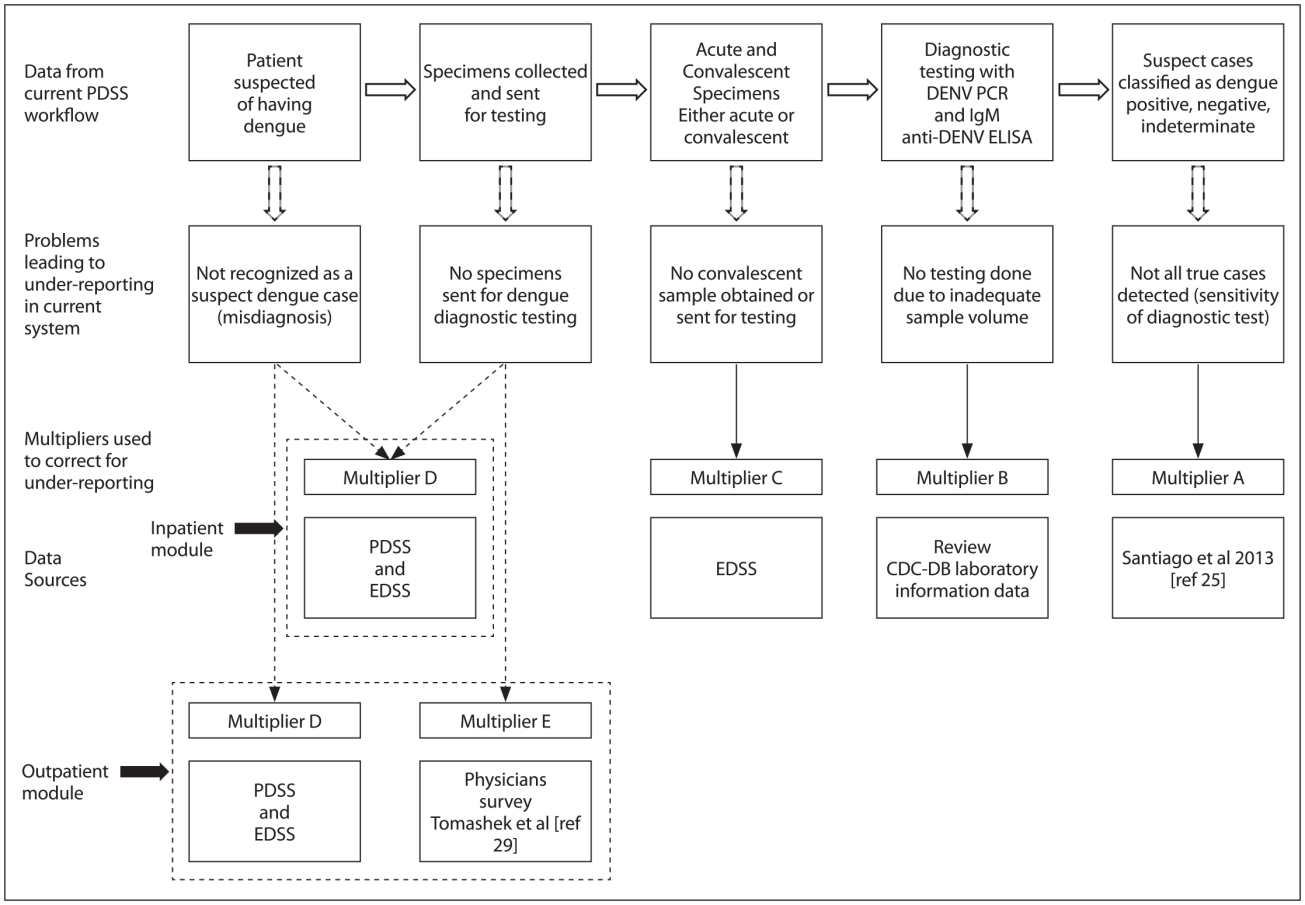
Schematic of model to estimate multipliers to correct for under-reporting of outpatient and hospitalized dengue cases. Notes; Multiplier D (only) is used for the inpatient module. Multiplier D and Multiplier E are used for the outpatient module. See text for further details. PDSS = Passive dengue surveillance system. EDSS = Enhanced dengue surveillance system. CDC-DB = U.S. Centers for Disease Control and Prevention, Dengue Branch (stationed in San Juan, Puerto Rico).

We estimated the degree of under-reporting of medically attended dengue cases in Puerto Rico by building a spreadsheet-based probability (Monte Carlo) multiplier model. The probability simulations were run using a spreadsheet add-in program (@Risk 7.0, Palisade, Ithaca, NY). We adapted models used to calculate the under-recognized impact of foodborne illness and pandemic influenza in the United States [19, 20]. We used data from two different surveillance systems in Puerto Rico: the Passive Dengue Surveillance System (PDSS), and Enhanced Dengue Surveillance System (EDSS) (see description under Data Sources). The model had separate modules for inpatients and outpatients (Fig 1). Using our model, for each module we calculated adjustments of the medically attended dengue case counts to account for five factors that could affect the degree of underreporting. These factors are: the sensitivity of the dengue diagnostic tests (Multiplier A); the number of submitted specimens not tested due to inadequate volume or incomplete information (used to derive Multiplier B); an estimate of the proportion of indeterminate dengue diagnostic test results considered as positives (Multiplier C); and differences in rates of reported laboratory-positive dengue cases between PDSS and the EDSS (Multiplier D). In the outpatient module, we also adjusted for the less than 100% submissions from medical providers of diagnostic specimens collected from suspected cases (Multiplier E; Fig 1). We list the values for each multiplier in Table 1 (see later for full description).

**Table 1:** Input parameters to estimate multipliers to correct for under-reporting in Puerto Rico (2005–2010).

Multiplier	Probability distribution	Parameter values (ranges)		Sources
		Hospitalized	Outpatient	
Multiplier A; Sensitivity of dengue diagnostic tests	Triangular for PCR-based test	Low:0.93 Most likely:0.98 High:0.99		[25]
	Triangular for IgM test	Low:0.5159 Most likely:0.5568 High:0.5970		[28]
Multiplier B: 1—proportion of suspect cases not tested due to inadequate specimen volume or incomplete information^b^	None	1	1	Passive and Enhanced Dengue Surveillance Systems
Multiplier C: Estimated proportion of cases with indeterminate diagnostic test results that would actually be positive	Triangular (MA Sub-model)^a^	Low:0.44 Most likely:0.67 High:0.77	Low:0.16 Most likely:0.44 High:0.74	CDC Dengue Branch
	Triangular (DO sub-model)^a^	Low:0.11 Most likely:0.65 High:0.78	Low:0.0 Most likely:0.3 High: 1	
Multiplier D: Difference between PDSS and EDSS in number of reported, laboratory-positive cases^c^	Uniform (MA sub-model)^a^	1.63–4.40	11.87–35.33	Passive and Enhanced Dengue Surveillance Systems
	Uniform (DO sub-model)^a^	1	2.46–11.22	
Multiplier E: Estimated proportion of outpatient suspect cases not reported to PDSS^c^^,^^d^	Uniform	Low:0.51 High:0.62		[29]

a: MA sub-model = “Medically Attended (MA),” which includes all patients who either had a completed Dengue Case Information Form (DCIF), or had some indication in their medical records (such as specimens sent to a laboratory for dengue testing) as potentially having a clinical case of dengue. In the second sub-model, labeled “DCIF Only (DO),” we included only those patients (in or out) definitively recorded as potential dengue case on a DCIF. See text for further details.

b: Multiplier B is set at 1 means that it is assumed that there are no cases with inadequate specimen volume that would prevent laboratory testing.

c: PDSS—Passive Dengue Surveillance System; EDSS—Enhanced Dengue Surveillance Systems

d: Multiplier E pertains only to outpatients. See text for additional details.

We further allowed for the fact that two different laboratory tests are used to test serum specimens from patients suspected of having dengue (see later for details). We corrected for different probabilities of false negative readings (i.e., which causes underreporting) associated with each type of test. For those patients tested using both tests, if one test result was positive and the other negative, we considered the patient as being “positive.” We used, to obtain corrected estimates of the number of medically attended cases, the previously described multipliers in the following general formulae:

Corrected hospitalized positive cases_Year X, Test Y_ = Number of reported hospitalized positive cases_YearX, TestY_ x ((1/Multiplier A) x (1/Multiplier B) x (Multiplier C) x Multiplier D)

Corrected outpatient cases_Year X, Test Y_ = Number of reported outpatient positive cases_YearX, TestY_ x ((1/Multiplier A) x (1/Multiplier B) x (Multiplier C) x Multiplier D x (1/Multiplier E))

Where Year X refers to the number of reported cases in a given year, and Test Y refers to the type of laboratory-based test (see later for details). The value for Multiplier A depends upon which test was used, while the values for Multipliers C and D depended upon which patient classification/ (initiation) sub-model was used (Table 1 and see later for details).

We defined indeterminate cases as specimens from both hospitalized and outpatient medically attended cases that had either no detected dengue RNA and/or no available specimen collected a week or later after illness onset to test for presence of anti-dengue virus antibodies. We used Multiplier C to estimate the percentage of indeterminate specimens that would likely have tested positive if they had sufficient dengue RNA and/ or had been collected a week after illness onset. We calculated the value of Multiplier C using data from CDC-DB databases indicating the percentage of submitted specimens (with sufficient serum to allow complete testing) that tested positive from both inpatients and outpatients (see details later). Given this methodology for indeterminate specimens (only), we set Multipliers A and B each to a value of 1. The remainder of the calculations to determine the corrected number of positives for the indeterminate specimens was the same as for the other specimens (i.e, used Multipliers D for inpatient specimens, and Multipliers D and E for outpatient specimens–see earlier). To clarify, for specimens with sufficient dengue RNA and/ or had been collected more than a week after illness onset, Multiplier C was set to 1 (i.e, no correction for indeterminate cases).

#### Calculating overall multiplier

We calculated the overall multipliers, to correct for under-reporting due to the total effect of all five factors (i.e., Multipliers A–E), as follows:

For either hospitalized or outpatient:

Overall multiplier to correct for underreporting _Year X_ = (sum of corrected estimates of positive cases for each test including indeterminate)_Year X_ / Number of reported clinically attended cases_Year X_

#### Year-to-year differences

Using the above methodology will result in different annual estimates of overall multipliers. This is because the corrected number of medically attended dengue cases will depend upon the relative portions of specimens tested using each test, and the proportion of specimens considered indeterminate (Supplementary Material Table A1 and Table A2). In actuality, these proportions changed each year in Puerto Rico.

#### Including probability

Because of uncertainty regarding the accuracy of each multiplier, we used available data to define probability distributions for each multiplier (Table 1; Supplementary Material Tables A3 –A5). We then used these distributions to calculate, for each year and test (as per the formulae, above), 10,000 iterations of the model, using the Monte Carlo methodology. In this methodology, for each iteration, the software randomly samples each probability distribution, and calculates the resultant number of corrected medically attended cases. From the 10,000 iterations for each year, we obtained a mean, standard deviation (SD), and 95% confidence interval of the overall multipliers (i.e., the range between the 2.5% and 97.5% confidence estimates).

There may be situations in which it may be difficult to obtain sufficient data to construct probability distributions for each multiplier. We note that, in such situations, our methodology can be applied using only point estimates (single values) for each multiplier.

#### Patient classification: Two sub-models

In addition to the inpatient and outpatient modules, we created two sub-models by using two definitions to classify both inpatients and outpatients. This is because not all patients suspected as having dengue, or even after laboratory conformation of dengue, had a completed Dengue Case Investigation Form (DCIF) used to report suspect dengue cases to the PDSS. We therefore defined the first sub-model, labeled “Medically Attended (MA),” to include all patients who either had a completed DCIF, or had some indication in their medical records (such as specimens sent to a laboratory for dengue testing) as potentially having a clinical case of dengue. In the second sub-model, labeled “DCIF Only (DO),” we included only those patients (in or out) definitively recorded as potential dengue case on a DCIF. The MA sub-model is the most permissive, maximizing the number of patients with potential clinical dengue. The DO sub-model is more restrictive, requiring completed documentation (the DCIF) indicating why a patient was suspected of having dengue, and recording results from laboratory tests.

### Data sources

#### Dengue surveillance systems

Passive Dengue Surveillance System (PDSS): This passive surveillance system captures data on suspected dengue cases from clinicians from all regions of Puerto Rico [21, 22]. The defining criterion for a patient to be entered into the PDSS is that the attending clinician classifies that patient as having “suspected dengue.” During the study period, CDC-DB supported the PDSS with laboratory-based dengue diagnostic testing upon submission of a serum specimen and a completed DCIF form with demographic and clinical information [5,7, 23,24]. Patient data and diagnostic test results were entered into a secure database for case reporting and analysis of epidemiologic trends.

Enhanced Dengue Surveillance System (EDSS): Two sites for EDSS were established in the southeastern Puerto Rico municipalities of Patillas and Guayama in 2005 and 2009, respectively. The Patillas site is a community health center that serves outpatients only, and the Guayama site is a hospital-based system that serves both outpatients and inpatients [5, 24]. In our model, we used only the inpatient data from Guayama (data available from June 2009–December 2010; Supplementary Material Table A3). The Patillas health center provides primary care services for nearly 90% of the residents of that municipality (24) (data available from June 2005–May 2010; Supplementary Material Tables A4 and A5). Surveillance at both sites was ‘enhanced’ by educating clinicians and patients about dengue the need to collect adequate serum specimens, and by placing on-site project staff at each site. These project staff ensured that health care providers completed the DCIF, obtained and sent specimens. The staff also validated data accuracy by comparing the DCIF with the patient’s medical chart. Suspected dengue cases at both sites were clinically ill persons with reported or documented fever of ≥ 38°C (100.5°F) lasting fewer than 7 days, and diagnosis and treatment with either two or more of the following symptoms or signs—headache, rash, eye pain, myalgia, arthralgia, hypotension, hemorrhage, hemoconcentration, or thrombocytopenia—or, with a dengue diagnosis suspected by the physician for some other reason. We note that, due to the different cases definitions in the two surveillance systems, there is not a complete concurrence in which dengue patients were entered into the two systems. We know of no way to correct this (see Discussion section).

#### Rates per unit population

To calculate values of Multiplier D, we calculated the rates of inpatients per 1,000 population using the PDSS and EDSS reported inpatients in Guyama (2006 population of 45,524; Supplementary Material Table A3). For rates of outpatients per 1,000 population, we used the PDSS and EDSS reported outpatients in Patillas (2006 population of 19,871; Supplementary Material Tables A4 and A5) (details later).

To calculate relevant rates per 1,000 general population for all of Puerto Rico, we used PDSS-recorded cases of dengue from all of Puerto Rico, rather than just the municipalities of Patillas and Guayama. The number, and thus rates per general population, of PDSS-recorded cases in Patillas and Guayama, were often small and displayed great variability (Supplementary Material Tables A3-A5). Thus, we elected to use PDSS data from the whole island to establish more reliable population-based rates of dengue. We calculated rates for all of Puerto Rico for inpatient and outpatient medically attended dengue patients, with and without the overall multipliers, using population estimates of 3,808,610 persons (2006 population). There is, however, geographic variability in dengue incidence in different regions and municipalities of Puerto Rico [8], as well as potential differences in health care seeking behaviors (see Discussion).

#### Dengue diagnostic testing

The same diagnostic testing algorithms were used for both PDSS and EDSS during the study period and have been previously described [5, 8, 24]. Serum collected from suspected dengue patients with acute illness (defined as ≤ 4 days of illness onset) were tested for evidence of dengue virus (DENV) RNA using a reverse transcription polymerase chain reaction (RT-PCR) test [25]. Serum from convalescing patients (defined as being ≥6 days after illness onset), suspected to have been ill from dengue were tested for evidence of anti-DENV immunoglobulin M (IgM) by antibody capture enzyme-linked immunosorbent assay (MAC ELISA) (DENV IgM) [26, 27]. Specimens collected from patients between five and six days after illness onset were tested by both DENV RNA and anti-DENV IgM ELISA. A laboratory-positive case for those patients was defined as being either DENV RNA positive or anti-DENV IgM positive in any specimen. Laboratory-negative cases had no anti-DENV IgM antibody detected in a convalescent specimen. Laboratory-indeterminate cases had no DENV RNA detected in an acute specimen and no available specimen collected > 6 days after illness onset to test for presence of anti-DENV IgM antibody.

#### Multiplier data

Multiplier A: Sensitivity and Specificity of Dengue tests:

Santiago et al [25] determined the sensitivity of dengue RT-PCR diagnostic tests used for PDSS and EDSS samples during the study period to be 98.0% (95%CI: 93.1–99.8) for positive cases (Table 1). Based on the data from Santiago et al [25], we used a specificity of 100%. The sensitivity for anti-DENV IgM ELISA was between 55.7% (95%CI: 51.6% to 59.7%) for positive cases [28]. We used for anti-DENV IgM ELISA, a specificity of 96.7% (95%CI: 94.7% to 98.4%). We used these values to construct triangular probability distributions for use in the probability analyses (Table 1). We tested, in our sensitivity analyses, the relative important of the values used for sensitivity and specificity.

Multiplier B: Proportion of suspect cases not tested:

We retrospectively examined data collected by CDC-DB and found that there were very few cases (<5%) where the submitted specimen(s) were unable to be tested. We therefore assumed no specimen was rejected due to inadequate volume or incomplete DCIF, and consequently used a multiplier of 1 (i.e., no correction). This assumption reduces the possibility of the final, overall multiplier over-estimating the actual number of cases.

Multiplier C: Estimated proportion of indeterminate cases that would be positive:

As described earlier, we used data from CDC-DB databases indicating the percentage of submitted specimens (with sufficient serum to allow complete testing) that tested positive from both inpatients and outpatients. We classified these patients into our two sub-models, MA and DO. For MA hospitalized patients, the probability of testing positive for dengue ranged from 0.44 to 0.77, with a median of 0.64 (Table 1). The values for MA outpatients and DO inpatients and outpatients are given in Table 1. We used these values to construct triangular probability distributions for use in the probability analyses (Table 1).

Multiplier D: Difference between PDSS and EDSS in rates of reported, laboratory-positive cases:

To derive an estimate of inpatient Multiplier D for inpatient cases, we compared between PDSS and EDSS rates of dengue inpatients per 1,000 population (Guyama) using 2009 and 2010 data (Table 1; Supplementary Material Table A3). We used these values to construct a uniform probability distribution for use in the probability analyses (Table 1). Note that we assumed, in the DO sub-model, that the EDSS recorded the same number of inpatients as the PDSS (i.e, Multiplier D for DO inpatients was set at 1 (Table 1).

For outpatient dengue cases, for both the MA and DO sub-models, we examined six years (2005–2010) of data from Patillas to calculate the relevant outpatient Multiplier D. For the MA population classification sub-model, we chose the multipliers from 2007 and 2010 to represent the range of possible values for Multiplier D (Supplementary Material Table A4). Not using the other, higher values (i.e., for 2006 and 2008) results in a lower, more conservative estimate of degree of PDSS-related under-reporting. For the DO population classification sub-model, because the range of calculated values was much smaller than for the MA sub-model, we chose the smallest and largest calculated values of Multiplier D (2010 and 2005, respectively–Supplementary Material Table A5). For both sub-models, we used these values to construct uniform probability distributions for use in the probability analyses (Table 1).

Multiplier E: Estimated proportion of outpatient suspect cases not reported to PDSS (Outpatients model only):

In 2007, CDC-DB conducted a survey of medical providers in Puerto Rico to ascertain their practices with respect to dengue reporting and management [29]. We calculated, from that survey, the 5^th^ and 95^th^ percentile confidence estimates of the proportion of physicians likely to report outpatient cases of dengue to PDSS. These estimates formed the lower and upper bounds of a uniform distribution used in the probability analyses (Table 1).

### Sensitivity analysis

We conducted a sensitivity analysis to determine which of the individual multipliers (A-D) were relatively the most important in determining the overall multiplier (as defined in the equations, earlier). We used the software to create a tornado graph of the 2010 inpatient multiplier (MA sub-model). The tornado graph plots out the partial correlation coefficients between the Overall Multiplier and the probability distributions defining the individual multipliers.

To examine the potential impact of laboratory-based false positive test results, we also altered the assumed specificity values of both the RT-PCR and IgM ELISA laboratory tests. We used the previously described data from 2010 concerning reported cases, inpatients and outpatients. We recalculated the multipliers after we reduced the specificity of both types of tests to an arbitrary 80%, well below the reported specificities of 100% and 96.7% (see earlier).

## Results

**Fig 2:**
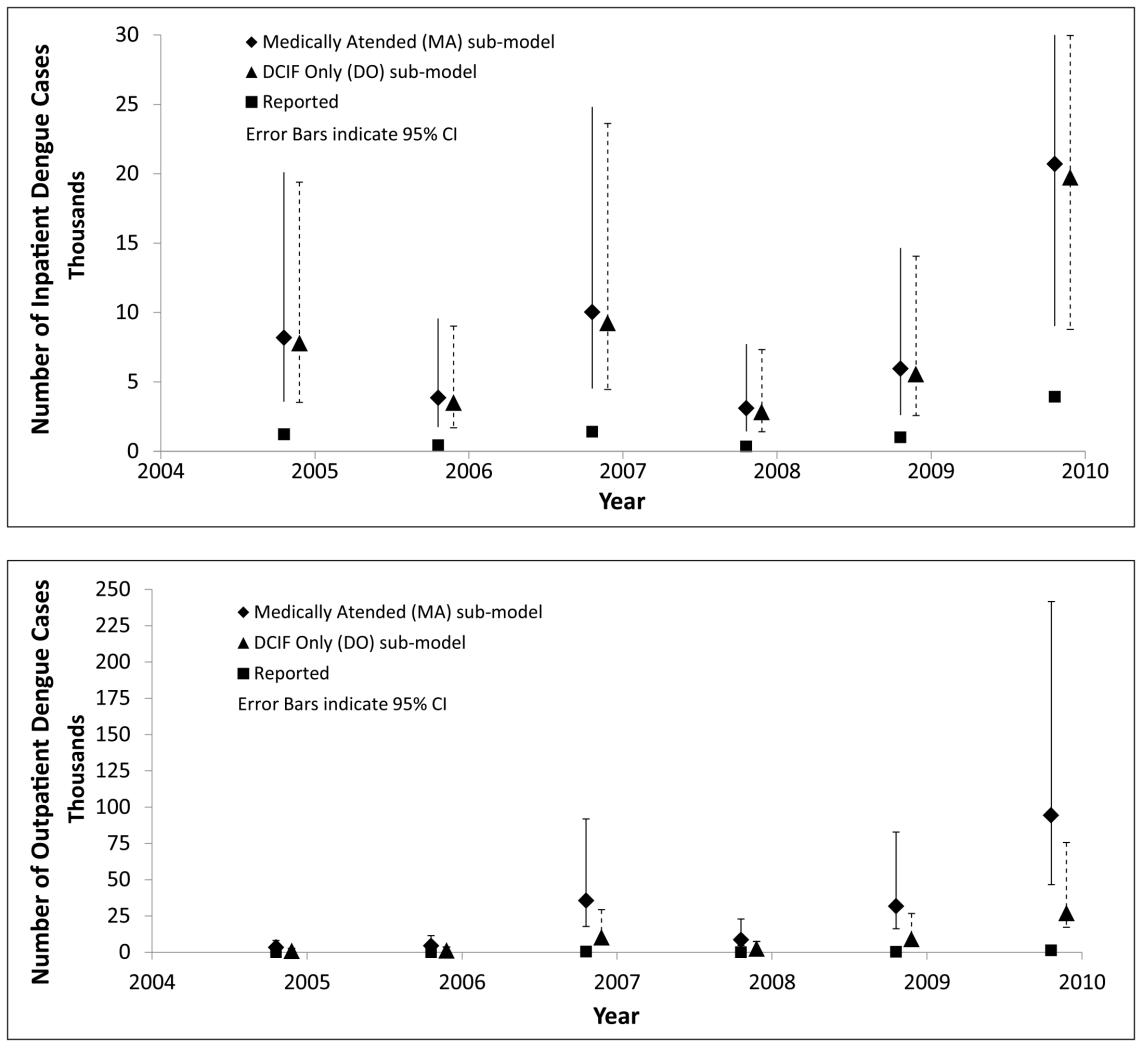
Reported and estimated number of clincial dengue cases in Puerto Rico, 2005–2010 (with 95% CI). Panel A: Reported and Estimated Dengue Inpatients; Panel B: Reported and Estimated Dengue Outpatients. Notes: MA = Medically Attended patient classification sub-model which includes all patients who either had a completed Dengue Case Information Form (DCIF), or had some indication in their medical records (such as specimens sent to a laboratory for dengue testing) as potentially having a clinical case of dengue. DO = In this patient classification sub-model, labeled “DCIF Only (DO),” we included only those patients (in or out) definitively recorded as potential dengue case on a DCIF. See text for further details. The 95% CI (confidence interval) is the range between the 2.5% and 97.5% confidence estimates.

The number of inpatients and outpatients reported with dengue showed a great deal of year-to-year variability, with 2010 having the most (Fig 2A and 2B: Supplementary Material Tables A1 and A2). In 2010, there was dengue epidemic on Puerto Rico [8]. The mean number inpatients, corrected for under-reporting, ranged from approximately 3,100 in 2008 to 20,700 in 2010 (Fig 2A). There were, excluding indeterminate cases, 361 and 3,941 reported dengue inpatients for those two years, respectively (Fig 2A; Supplementary Material Table A1). There was little difference between the estimates of corrected-for-under-reporting inpatients produced using the two patient classification sub-models (Fig 2A). The corrected estimates from both sub-models displayed large degrees of variability, with the greatest variability seen in the estimates for 2007 and 2010. In 2010, the 95% CI estimates of inpatients corrected for under-reporting ranged from approximately 11,000 to 30,000 inpatients (Fig 2A). For outpatients, there was greater variability between the estimates of corrected inpatients produced using the two patient classification sub-models (Fig 2B). The greatest variability occurred in correcting the reported dengue outpatients for 2010, when 1,266 dengue outpatients were reported. The MA patient classification sub-model produced a mean estimate for 2010 of approximately 94,400 (95% CI range: 48,100–147,700) dengue outpatients. The DO sub-model, in comparison, produced a mean estimate for 2010 of approximately 27,000 (95% CI range: 10,000–49,000) dengue outpatients.

For the MA patient classification sub-model, the calculated overall multipliers for inpatients ranged from 5.25 (SD 1.4) for 2010, to 8.66 (SD 2.35) for 2008 (Table 2). The multipliers for inpatients using the DO sub-model, ranged from 5.01 (SD 1.37) in 2010, to 7.85 (SD 2.35) in 2008 (Table 2). The estimated multiplier for outpatient cases using the MA sub-model ranges from approximately 75 (SD 22), for 2010, to115 (SD 37) for 2008 (Table 3). Using the DO sub-model, the multiplier ranges from approximately 22 (SD 9) for 2010, to 33 (SD 15) for 2008) (Table 3).

**Table 2:** Estimates of dengue overall multiplier for inpatients in Puerto Rico: 2005 to 2010^a^.

Year	MA sub-model: all hospitalized suspect dengue patients^b^				DO sub-model: definitively recorded hospitalized dengue patients^b^			
	Mean	Std Dev	2.5th percentile	97.5th percentile	Mean	Std Dev	2.5th percentile	97.5th percentile
**2005**	6.64	1.77	3.74	9.63	6.32	1.73	3.49	9.42
**2006**	8.63	2.35	4.77	12.73	7.86	2.32	4.07	12.29
**2007**	7.04	1.89	3.95	10.34	6.48	1.87	3.39	10.07
**2008**	8.66	2.35	4.80	12.76	7.85	2.35	3.98	12.45
**2009**	5.87	1.57	3.30	8.56	5.48	1.54	2.97	8.36
**2010**	5.25	1.40	2.96	7.62	5.01	1.37	2.78	7.44

a: Calculated, for inpatient only, using data from 2009 and 2010. See main text and Supplementary Material Table A3.

b: MA sub-model = “Medically Attended (MA),” which includes all patients who either had a completed Dengue Case Information Form (DCIF), or had some indication in their medical records (such as specimens sent to a laboratory for dengue testing) as potentially having a clinical case of dengue. In the second sub-model, labeled “DCIF Only (DO),” we included only those patients (in or out) definitively recorded as potential dengue case on a DCIF. See text for further details.

**Table 3:** Estimates of dengue overall multiplier for outpatients in Puerto Rico: 2005 to 2010.

	MA^a^				DO^a^			
Year	Mean	Std Dev	2.5th percentile	97.5th percentile	Mean	Std Dev	2.5th percentile	97.5th percentile
**2005**	84.46	25.11	43.55	129.67	24.33	9.47	9.24	42.48
**2006**	95.36	30.21	46.58	155.34	27.26	11.98	9.16	53.35
**2007**	80.75	24.64	40.75	127.78	23.19	9.67	8.20	43.60
**2008**	115.06	37.29	55.31	191.61	32.85	15.00	10.60	66.91
**2009**	89.10	27.96	43.84	143.70	25.49	10.96	8.90	49.50
**2010**	74.55	22.43	37.99	116.68	21.41	8.59	7.93	38.74

a: MA sub-model = “Medically Attended (MA),” which includes all patients who either had a completed Dengue Case Information Form (DCIF), or had some indication in their medical records (such as specimens sent to a laboratory for dengue testing) as potentially having a clinical case of dengue. In the second sub-model, labeled “DCIF Only (DO)” we included only those patients (in or out) definitively recorded as potential dengue case on a DCIF. See text for further details.

For both inpatients and outpatients, the rates per 1,000 general population of reported inpatient dengue cases increased from 0.32/1,000 (2005) to 1.03 (2010) (Table 4). Rates of reported dengue outpatients had an even larger increase, from 0.01/1,000 (2005) to 0.10/ 1,000 (2010)(Table 4). The corrected rates for inpatients, averaged over 6 years, ranged from 2.13/ 1,000 (DO sub-model) to 2.27/1,000 (MA sub-model) (Table 4). The 6 year average corrected rate for dengue outpatients ranged from 2.24/ 1,000 (DO sub-model) to 7.79/1,000 (MA sub-model) (Table 4).

**Table 4:** Reported and estimated rate of medically attended dengue per 1,000 general population in Puerto Rico (2005 to 2010).

Year	Rates per 1,000 general population of medically attended dengue cases^b^					
	Inpatients			Outpatients		
	Reported dengue	Rates of reported dengue corrected for under-reporting^b^		Reported dengue	Rates of reported dengue corrected for under-reporting^b^	
		MA sub-modela	DO sub-modela		MA sub-model^a^	DO sub-model^a^
2005	0.32	2.15	2.05	0.01	0.86	0.25
2006	0.12	1.02	0.92	0.01	1.15	0.33
2007	0.37	2.64	2.43	0.12	9.35	2.69
2008	0.09	0.82	0.74	0.02	2.27	0.65
2009	0.27	1.57	1.46	0.09	8.33	2.38
2010	1.03	5.44	5.18	0.33	24.78	7.12
All Years^c^	0.37	2.27	2.13	0.10	7.79	2.24

a: MA sub-model = “Medically Attended (MA),” which includes all patients who either had a completed Dengue Case Information Form (DCIF), or had some indication in their medical records (such as specimens sent to a laboratory for dengue testing) as potentially having a clinical case of dengue. In the second sub-model, labeled “DCIF Only (DO),” we included only those patients (in or out) definitively recorded as potential dengue case on a DCIF. See text for further details.

b; Mean of the 10,000 iterations from the model.

c: Mean of dengue rates, reported and corrected, for years 2005–2010.

### Sensitivity analysis

**Fig 3:**
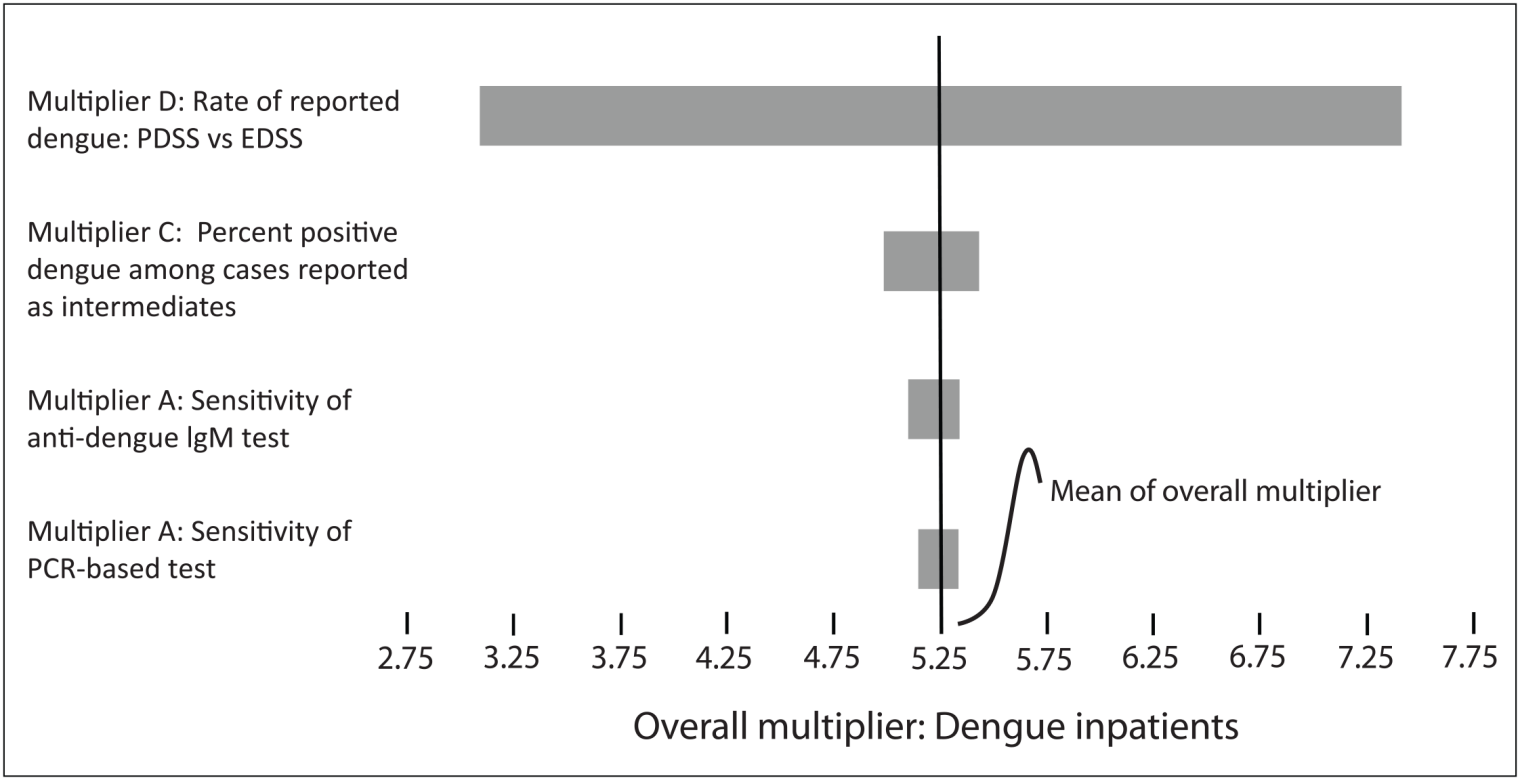
Sensitivity analysis: Relative importance of individual multipliers in calculating the overall multiplier for reported dengue inpatients: Puerto Rico, 2010. Notes: a: Graph plots relative importance of the individual multipliers used to calculate the overall multiplier. The wider the plotted range (i.e., bar), the greater the change in the overall multiplier. b: Results calculated for MA = Medically Attended patient classification sub-model which includes all patients who either had a completed Dengue Case Information Form (DCIF), or had some indication in their medical records (such as specimens sent to a laboratory for dengue testing) as potentially having a clinical case of dengue. PDSS = Passive dengue surveillance system. EDSS = Enhance dengue surveillance system. IgM = Immunoglobulin M. Test indicates presence of dengue IgM antibodies using an antibody capture enzyme-linked immunosorbent assay. PCR = Polymerase chain reaction. Test indicates evidence of dengue RNA using by a Reverse transcription polymerase chain reaction (RT-PCR) test. See main text for detailed description of individual multipliers and how they are used to calculate the overall multiplier.

The tornado graph shows that the relatively most important individual multiplier is rate of reported dengue: PDSS vs EDSS (Multiplier D) (Fig 3). The next most important individual multiplier was Multiplier C (Percent positive dengue among cases reported as intermediates). However, the partial correlation coefficient between Multiplier C and the overall Multiplier was much smaller than that for Multiplier D, indicating a relatively small effect between changes in Multiplier C and the Overall Multiplier.

When we tested for the potential impact of false-positive test result on our estimates of multipliers, we reduced the specificities of the laboratory-based test to 80% (from the baseline values of 100% for RT-PCR and a mean of 96.7% for the anti-DENV IgM ELISA). For outpatients, the estimated multipliers for 2010 increased from 74.55 for the MA model and 21.41 for the DO model (Table 3) to 76.27 and 21.92, respectively. For inpatients, the estimated multipliers for 2010 increased from 5.25 for the MA model, and 5.01 for the DO model (Table 2) to 5.35 and 5.10, respectively. We concluded that all of these results illustrate that false positive test results are unlikely to increase notably the size of our estimated multipliers.

## Discussion

We estimated that a multiplication factor of between 5 (for 2010 data) to 9 (for 2006 data) must be used to correct for under-reporting the number of laboratory-positive dengue inpatients reported to the Puerto Rican PDSS. Multiplication factors of between 21 (for 2010 data) to 115 (for 2008 data) must be used to correct for under-reporting of laboratory-positive dengue outpatients. We also estimated that the mean annual rate of medically attended dengue in Puerto Rico to be between 2.1 (for dengue inpatients) to 7.8 (for dengue outpatients) per 1,000 population. These estimated rates compare to the reported rates of 0.4 (dengue outpatients) to 0.1 (dengue inpatients) per 1,000 population. The estimates were most sensitive to the difference between PDSS and EDSS in numbers of reported, laboratory-positive cases, and how we identified potential clinical cases of dengue. Use of the more permissive Medically Attended (MA) classification resulted in notably higher estimates of overall multipliers for outpatient dengue cases than estimates obtained when using the more restrictive DCIF-Only (DO) patient classification.

Previously published estimates of multipliers for dengue inpatients ranged from 1.4–3.4 for four countries in the Americas (including Puerto Rico) and 1.8–2.5 for five South East Asian countries [17, 18]. In comparison, our estimates for dengue inpatients were higher, ranging from 5 to 9. Interestingly, our inpatient estimate is comparable with a multiplier found for laboratory-positive dengue deaths in Puerto Rico found using an enhanced surveillance system. In that study [32] the mortality rate was 2–3 times higher than detected previously under the passive surveillance system. One would expect fatal cases to be more readily identifiable and reported than for inpatient dengue cases. For outpatients, the previously published estimates ranged between 1–28 for four countries in the Americas (including Puerto Rico) and 5.0–29.8 for Southeast Asian countries [17,18]. The upper end of that range is similar to our range of estimates of 21–33 for outpatient cases, which we calculated using the DO sub-model (Table 3). Our estimates from the outpatients MA sub-model, however, were notably higher, at 75–115 (Table 3). We are only aware of one study that had similar multiplier estimates for outpatient cases. That a study was from Malaysia, and was based on expert opinion that there were 65.6 additional cases for each identified outpatient case [18]. This wide variability of estimated underreporting indicates that there is little meaningful correlation between the relative amount of resources available to public health and clinical health care systems and the ability of such systems to accurate capture the number of outpatient and inpatient dengue case. The reason for such lack of definitive correlation remains to be explained.

This study has several limitations. We assumed that the rate of positivity among those with both acute and convalescent specimens was the same as those who had only an acute specimen submitted (i.e., indeterminate cases). We also assumed that the rate of dengue in EDSS areas (Guayama and Patillas) was representative for all of Puerto Rico, which may not be accurate. When calculating rates of dengue from the PDSS system, we used all cases reported in the PDSS system for the entire island of Puerto Rico. This method may mask a degree of heterogeneity in rates of cases on the island. Further, the health care seeking behaviors of patients for whom two serum specimens were submitted (one while acutely ill, another while convalescent, necessary for complete diagnosis using the IgM-based test) may be different than behaviors of those for whom had only one specimen submitted. Correcting for these limitations would require an analysis in nearby municipalities, with high and low incidence of dengue, or cases from within the EDSS municipalities that were not reported through the EDSS. The resources for such a level of surveillance were not available.

The estimated expansion factors for 2005–2010 were quite consistent year-to-year. Thus, we have no reason to believe that estimates of multipliers for later years would be notably different than those shown in Table 2. The EDSS in Patillas ended in 2012. Dengue inpatient EDSS in Guyama continued, and enhanced surveillance was implemented in 2012 at Saint Luke's Episcopal Hospital in Ponce municipality. However, following the arrival of chikungunya and Zika viruses to Puerto Rico in 2014 and 2016, respectively [30, 31], it is unclear how case reporting or rates of laboratory-confirmation of dengue may be affected. Additional investigation may therefore be necessary to define the rates of both under-reporting of clinically-apparent illness caused by these pathogens.

There is also the potential for correlation between some of the multipliers. For example, doctors that reported cases of dengue may work in areas where there were more laboratory-tested cases of febrile illnesses. There thus could be a negative correlation between Multipliers D and E. We do not have any evidence, however, that doctors who reported cases of dengue in the areas studied were more likely to be practicing in areas that have a higher likelihood of dengue cases among patients with acute febrile illnesses. In addition, in a given locale dengue incidence varies over time, due to the buildup of naturally acquired immunity in individuals and thus increased herd immunity. For example, towns and municipalities that were severely impacted in the 2007 dengue outbreak were less likely to be impacted in the 2010 dengue outbreak. Further, many of the physicians in Puerto Rico work at least part-time in large referral hospitals and see patients from several municipalities with varying dengue incidence that changes over time. Thus, doctors who report dengue are actually likely to encounter patients from a variety of locales with varying risks of acquiring dengue.

Finally, as mentioned earlier, there is not a complete concurrence in which dengue patients were entered into the two systems. We know of no way to correct this problem, or to correct for the problems related to having only two EDSS sites. These problems are not unique to Puerto Rico. Many countries that have passive dengue surveillance systems with non-specific case definitions, and have often lacked comprehensive dengue-based surveillance (14, 15, 18). We maintain that, even with these problems, the multipliers presented here are sufficient to allow public health officials to usefully correct for under-reporting.

This study augments existing evidence showing that reporting of severe cases of dengue, which are more likely to be admitted to hospitals, is more accurate than for less severe outpatient cases. Our results demonstrates the benefits of an enhanced dengue surveillance system, which can supplement passive dengue surveillance systems in countries with resource constraints. As noted earlier, in situations where there are insufficient data to construct probability distributions for each multiplier, our methodology can be applied using only point estimates (single values) for each multiplier. Our results illustrate the need for, and thus potential benefits of, frequently using our methodology to estimate the degree of under-reporting in passive dengue systems during epidemic and non-epidemic years.

## Disclaimer

The findings and conclusions in this report are those of the authors and do not necessarily represent the views of the Centers for Disease Control and Prevention

## Supporting information

S1 Text Supplementary material. (DOCX)
