# Supplementary material for: Estimating dengue under-reporting in Puerto Rico using a multiplier model
Source: PLoS Negl Trop Dis. 2018 Aug 6;12(8):e0006650. doi: 10.1371/journal.pntd.0006650 (PMC6095627; doi:10.1371/journal.pntd.0006650)
Supplement: S1 Text — (DOCX) [file pntd.0006650.s001.docx]

**SUPPLEMENTARY MATERIAL**

TITLE: Estimating dengue under-reporting in Puerto Rico using a multiplier model

AUTHORS: Manjunath B. Shankar^1,a^, Rosa L. Division of Rodríguez-Acosta^2,b^, Tyler M. Sharp^2^, Kay M. Tomashek^2,c^, Harold S. Margolis^2,d^, Martin I.Meltzer^1*^

Affiliations:

1. Division of Preparedness and Emerging Infections, National Center for Emerging and Zoonotic Infectious Diseases, Centers for Disease Control and Prevention, Atlanta, GA.

2. Dengue Branch, Division of Vector-Borne Diseases, National Center for Emerging and Zoonotic Infectious Diseases, Centers for Disease Control and Prevention, Puerto Rico.

Current addresses:

a. Current address: Department of Community Medicine, Care Convergence Center, Hyderabad, India.

b. Current address: Division of Safety Research, National Institute for Occupational Safety and Health, Centers for Disease Control and Prevention, Atlanta, GA.

c. Current address: National Institute of Allergy and Infectious Diseases, National Institutes of Health, Bethesda, MD.

d. Current address: Retired.

*CORRESPONDING AUTHOR: Martin I. Meltzer, Division of Preparedness and Emerging Infections, NCEZID, CDC. Mailstop C-18, 1600 Clifton Rd, Atlanta, GA, UNITED STATES, 30329. Ph: 404-639-7778; e-mail: MMeltzer@cdc.gov

ORCID numbers: Martin I. Meltzer: 0000-0002-7250-0472

KEY WORDS: Dengue; correcting under-reporting; multipliers; Puerto Rico;

**Table A1:** Numbers of positive test results by type of test and indeterminate tests for inpatients as reported to the Passive Dengue Surveillance System, Puerto Rico (2005-2010)

| Test result | Type of test^a^ | Year | | | | | |
| --- | --- | --- | --- | --- | --- | --- | --- |
|  |  | 2005 | 2006 | 2007 | 2008 | 2009 | 2010 |
|  | | Patients tested either < 4 days or > 6 days post symptom onset^a^ | | | | | |
| Positive | PCR | 21 | 19 | 104 | 14 | 80 | 298 |
|  | IgM | 69 | 31 | 87 | 25 | 18 | 161 |
|  | | Patients tested day 5 or day 6 post symptom onset^a^ | | | | | |
| Positive | PCR | 273 | 188 | 816 | 178 | 662 | 2319 |
|  | IgM | 873 | 210 | 421 | 144 | 256 | 1163 |
| Indeterminate^b^ | | 1142 | 1008 | 2332 | 848 | 1138 | 2816 |

a: Serum collected from suspected dengue patients with acute illness (defined as < 4 days of illness onset) were tested for evidence of dengue RNA using a reverse transcription polymerase chain reaction (PCR) test. Serum from convalescing patients (defined as being ≥6 days after illness onset), suspected to have been ill from dengue were, were tested for evidence of anti-dengue immunoglobulin M (IgM) by antibody capture enzyme-linked immunosorbent assay (MAC ELISA). Specimens collected between five and six days after illness onset were tested by both PCR and IgM. A laboratory-positive case was defined as being either dengue RNA positive or IgM positive in any specimen.

b: Laboratory-indeterminate cases had no RNA detected in an acute specimen and no available specimen > 6 days to test for presence of IgM.

**Table A2:** Numbers of positive test results by type of test and indeterminate tests for outpatients as reported to the Passive Dengue Surveillance System, Puerto Rico (2005-2010)

| Test result | Type of test^a^ | Year | | | | | |
| --- | --- | --- | --- | --- | --- | --- | --- |
|  |  | 2005 | 2006 | 2007 | 2008 | 2009 | 2010 |
|  | | Patients tested either < 4 days or > 6 days post symptom onset^a^ | | | | | |
| Positive | PCR | 0 | 2 | 37 | 1 | 18 | 83 |
|  | IgM | 2 | 0 | 19 | 2 | 3 | 29 |
|  | | Patients tested day 5 or day 6 post symptom onset^a^ | | | | | |
| Positive | PCR | 11 | 32 | 290 | 45 | 246 | 812 |
|  | IgM | 26 | 12 | 95 | 27 | 89 | 342 |
| Indeterminate^b^ | | 39 | 109 | 702 | 241 | 725 | 1515 |

a: Serum collected from suspected dengue patients with acute illness (defined as < 4 days of illness onset) were tested for evidence of dengue RNA using a reverse transcription polymerase chain reaction (PCR) test. Serum from convalescing patients (defined as being ≥6 days after illness onset), suspected to have been ill from dengue were, were tested for evidence of anti-dengue immunoglobulin M (IgM) by antibody capture enzyme-linked immunosorbent assay (MAC ELISA). Specimens collected between five and six days after illness onset were tested by both PCR and IgM. A laboratory-positive case was defined as being either dengue RNA positive or IgM positive in any specimen.

b: Laboratory-indeterminate cases had no RNA detected in an acute specimen and no available specimen > 6 days to test for presence of IgM.

**Table A3**: Calculating inpatient multiplier D: Ratio of rates of dengue inpatients in Guyama for the Medically Attended (MA) patient classification sub-model^c, d,e^

| Year | Rate of dengue inpatients per 1,000 population in Guyama: EDSS^a,b^ | Rate of dengue inpatients per 1,000 population: PDSS^a^ | Inpatient multiplier D: Dengue rate EDSS/Dengue rate PDSS^a^ |
| --- | --- | --- | --- |
| 2009 | 0.97 | 0.22 | 4.40 |
| 2010 | 1.36 | 0.83 | 1.63 |

a: PDSS – Passive dengue surveillance system; EDSS - Enhanced dengue surveillance system.

b: EDSS rates of inpatient was measured in Guyama at a hospital with a known “catchment area” population.

c: “Medically Attended (MA),” which includes all patients who either had a completed Dengue Case Information Form (DCIF), or had some indication in their medical records (such as specimens sent to a laboratory for dengue testing) as potentially having a clinical case of dengue.

d: For the other patient classification sub-model, DCIF Only (DO), we assumed that there was no difference between the PDSS and EDSS in reporting inpatients. Thus, for the DO sub-model, the inpatient multiplier was set at 1. See main text for further details.

e. Guyama population of 45,524 (2006 data).

**Table A4:** Calculating range of outpatient multiplier D: Ratio of rates of dengue outpatients in Patillas for the Medically Attended (MA) patient classification sub-model^c,e^.

| Year | Rate of dengue outpatients per 1,000 populations EDSS^b^ | Rate of dengue outpatients per 1,000 population: PDSS^a^ | Multiplier D: Dengue rate EDSS/Dengue Rate PDSS |
| --- | --- | --- | --- |
| 2005 | 7 | 0 | NA |
| 2006 | 1.79 | 0.01 | 179 |
| 2007 | 3.18 | 0.09 | **35.33^d^** |
| 2008 | 1.14 | 0.01 | 114 |
| 2009 | 0.95 | 0.08 | **11.88^d^** |
| 2010 | 1.35 | 0.27 | 5 |

a: PDSS – Passive dengue surveillance system; EDSS - Enhanced dengue surveillance system.

b: EDSS rates of outpatient were measured at a Patillas outpatient clinic with a known “catchment area” population.

c: “Medically Attended (MA),” which includes all patients who either had a completed Dengue Case Information Form (DCIF), or had some indication in their medical records (such as specimens sent to a laboratory for dengue testing) as potentially having a clinical case of dengue.

d: The Multipliers of PDSS to EDSS from these two years (2007 and 2010) were chosen to represent the range of possible values for Multiplier D (for the MA sub-model). Not using the other, higher, values (for 2006 and 2008) results in a lower, more conservative estimate of degree of under-reporting.

e: Patillas population 19,871 (2006).

**Table A5:** Calculating range of outpatient multiplier D: Ratio of rates of dengue outpatients in Patillas for the DCIF-only (DO) patient classification sub-model^c,e^.

| Year | Rate of dengue outpatients per 1,000 populations EDSS^b^ | Rate of dengue outpatients per 1,000 population: PDSS^a^ | Multiplier D: Dengue rate EDSS/Dengue Rate PDSS |
| --- | --- | --- | --- |
| 2005 | 7.68 | 0.68 | **11.23^d^** |
| 2006 | 2.08 | 0.20 | 10.45 |
| 2007 | 4.15 | 0.88 | 4.69 |
| 2008 | 1.50 | 0.22 | 6.79 |
| 2009 | 3.74 | 0.65 | 5.71 |
| 2010 | 6.85 | 2.78 | **2.46^d^** |

a: PDSS – Passive dengue surveillance system; EDSS - Enhanced dengue surveillance system.

b: EDSS rates of outpatient were measured at a Patillas outpatient clinic with a known “catchment area” population.

c: “DCIF-only (DO)” patient sub-classification model: This sub-model included only those patients definitively recorded as a potential dengue case on a Dengue Case Information Form (DCIF).

d: The Multipliers of PDSS to EDSS from these two years (2005 and 2010) were chosen to represent the range of possible values for Multiplier D (for the DO sub-model).

e: Patillas population 19,871 (2006).
